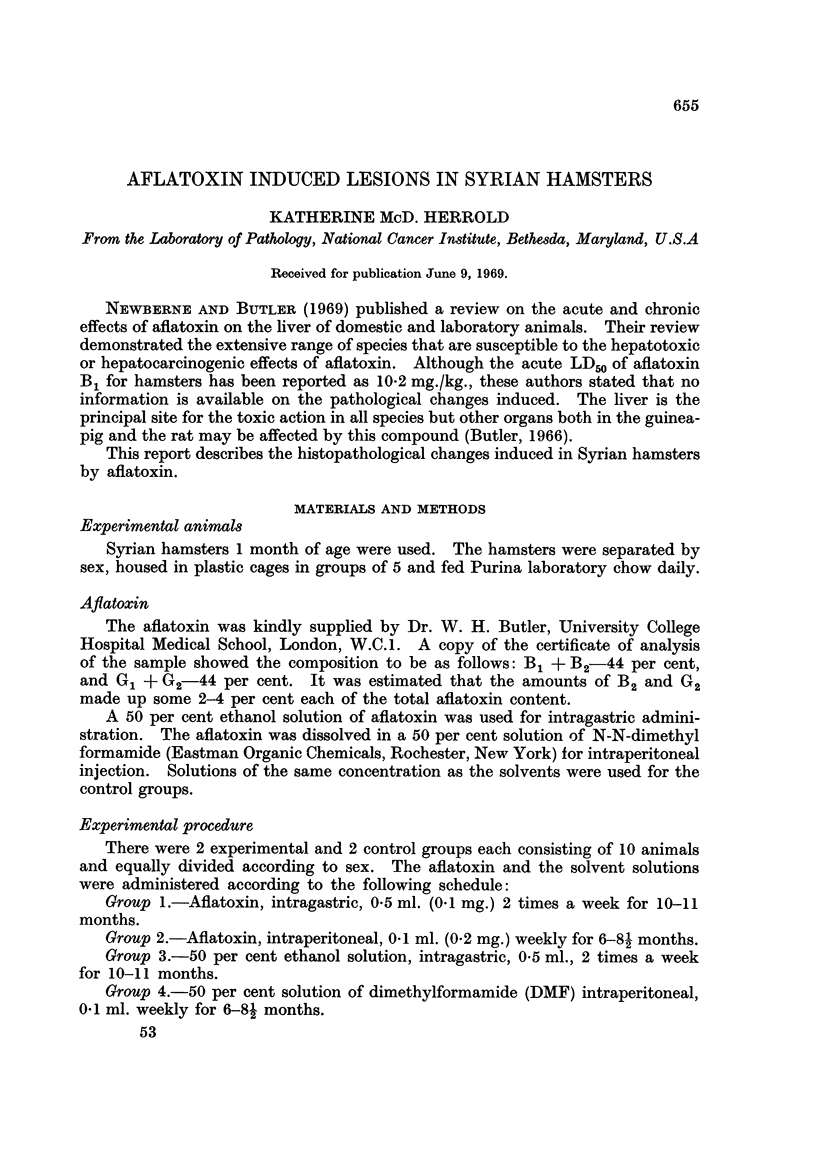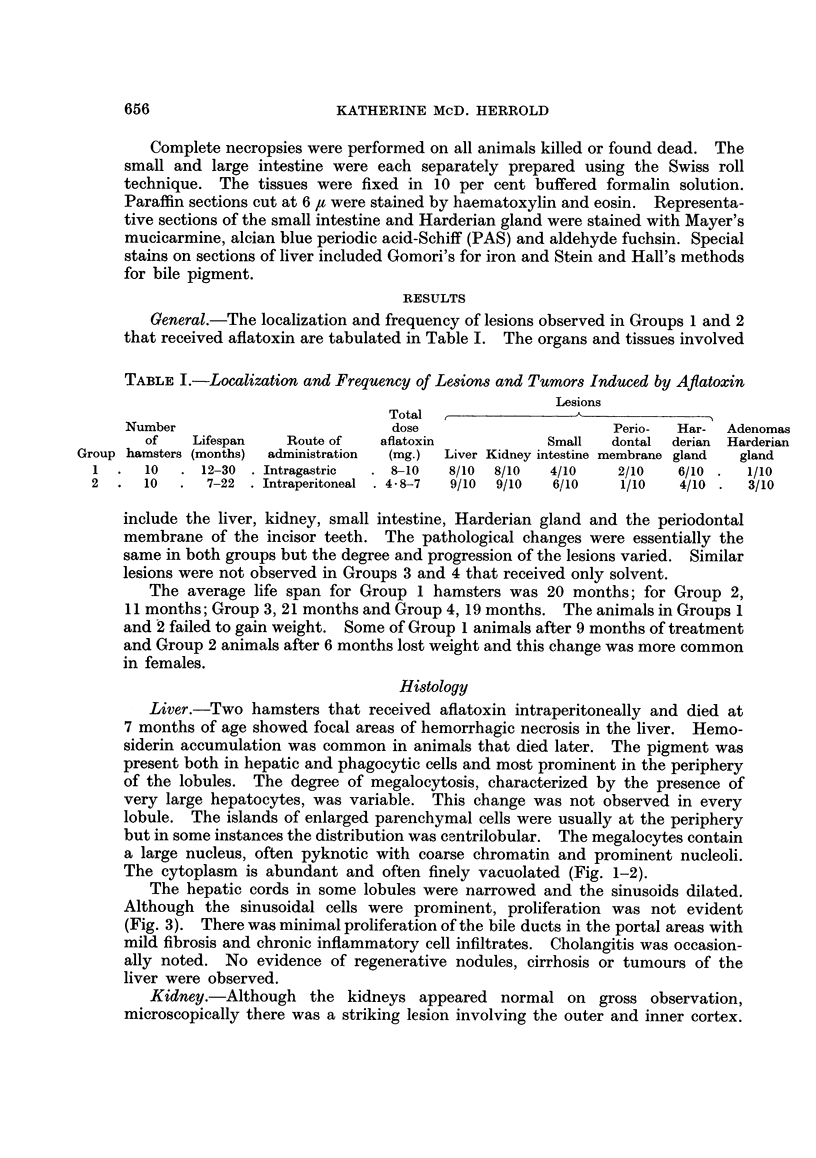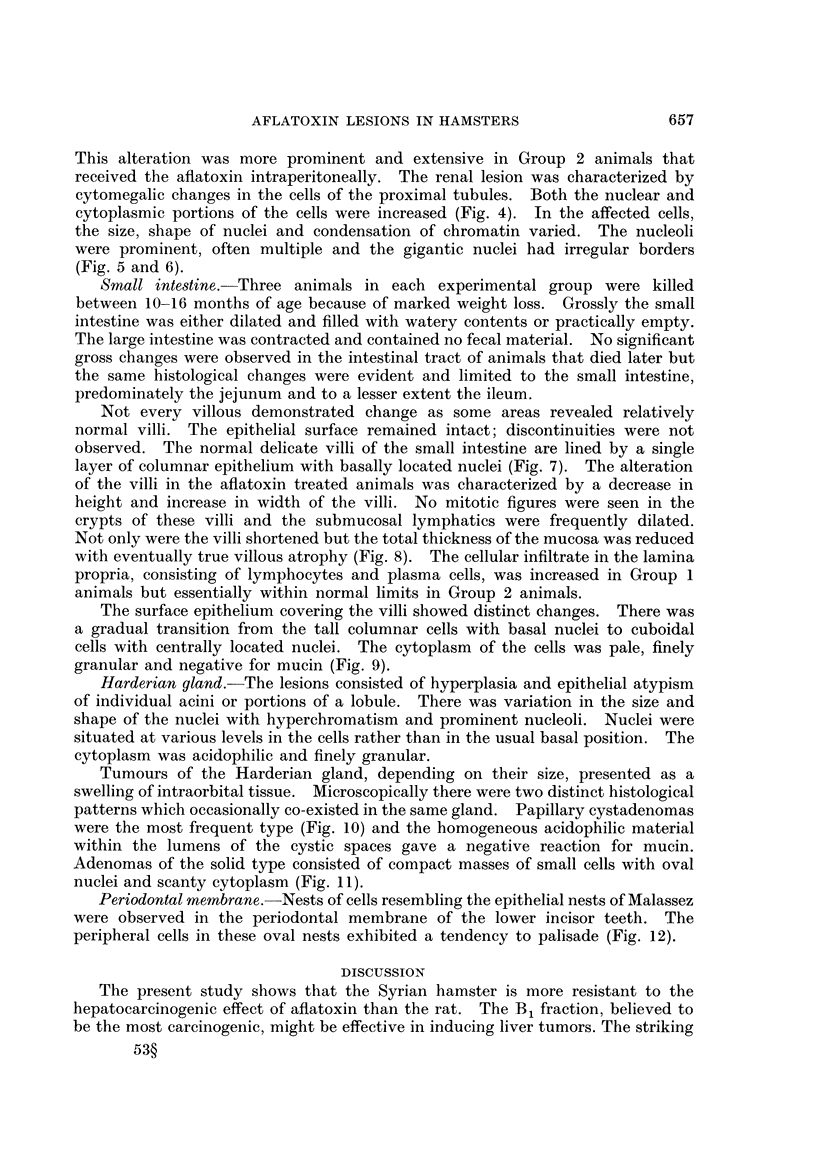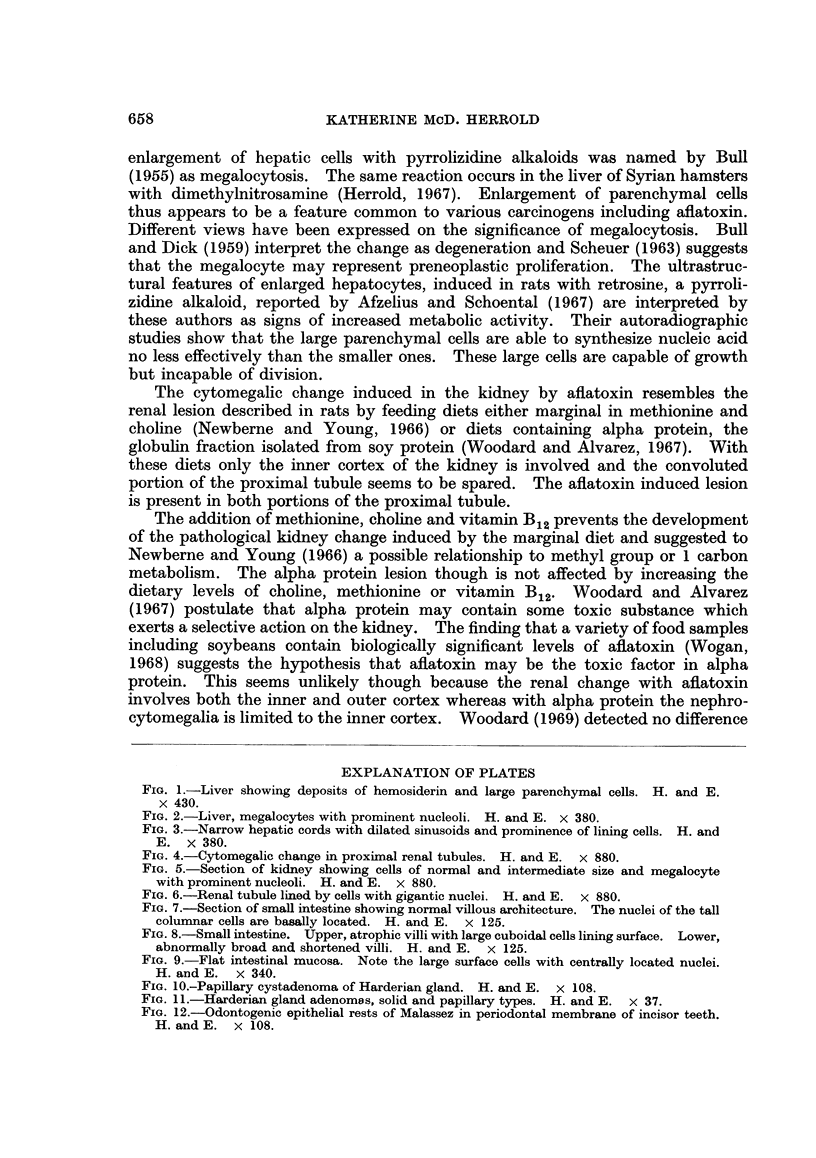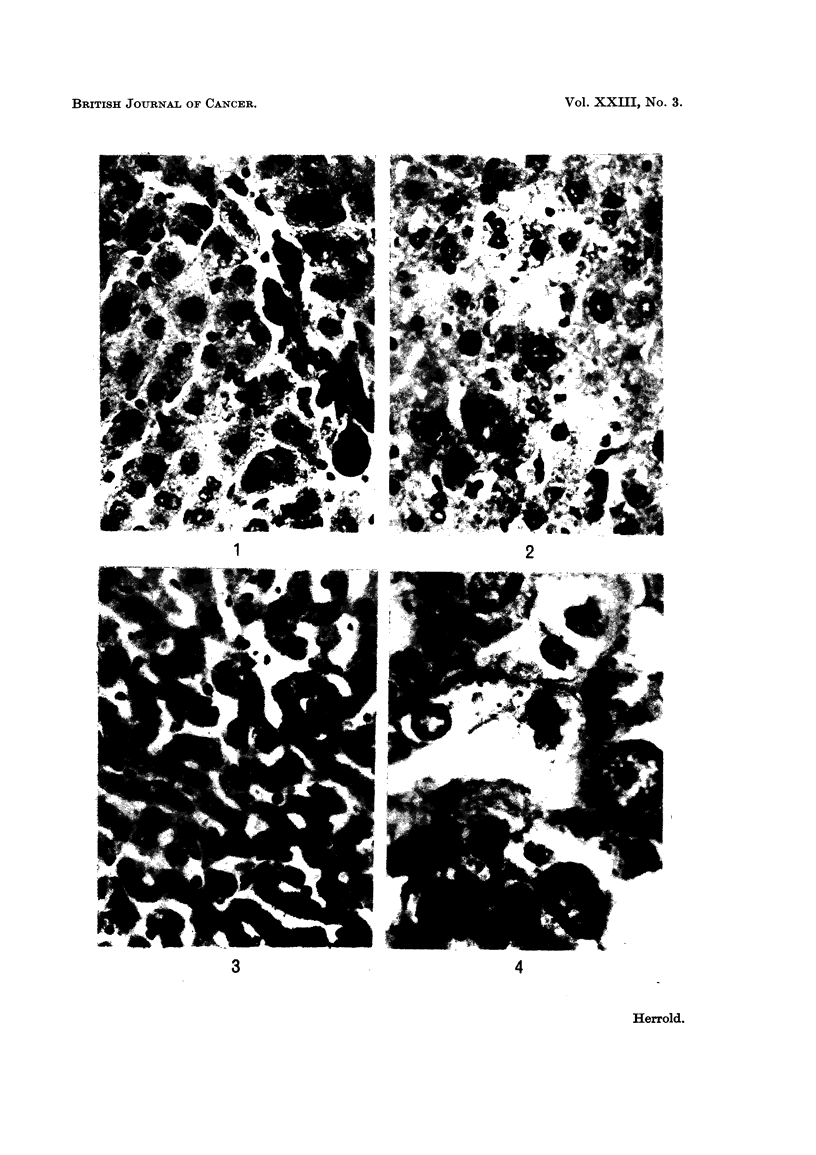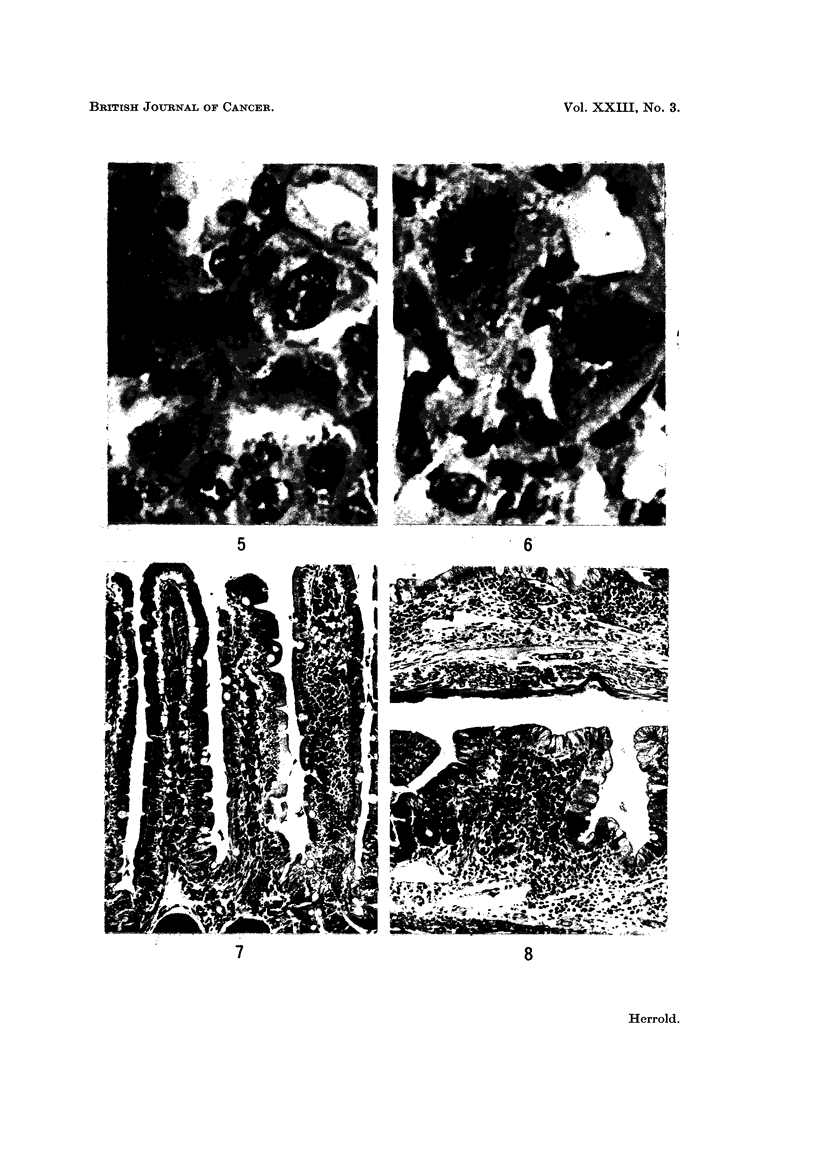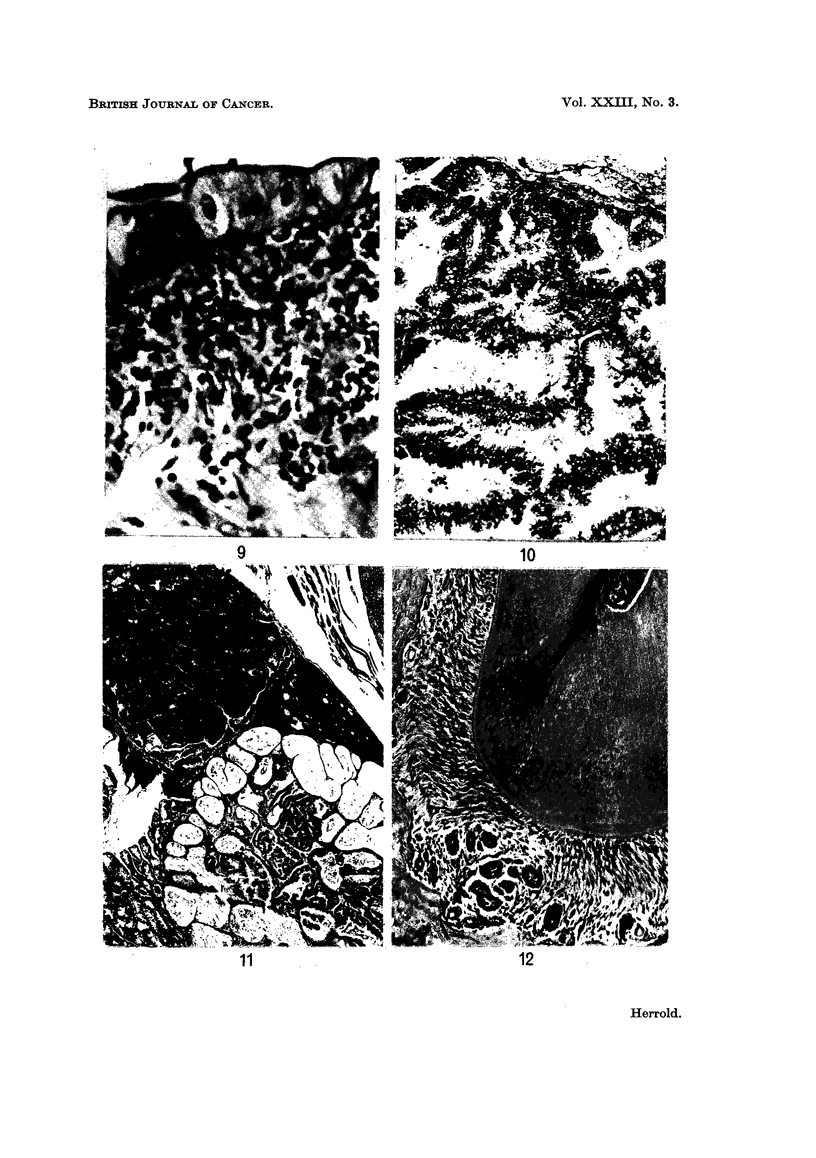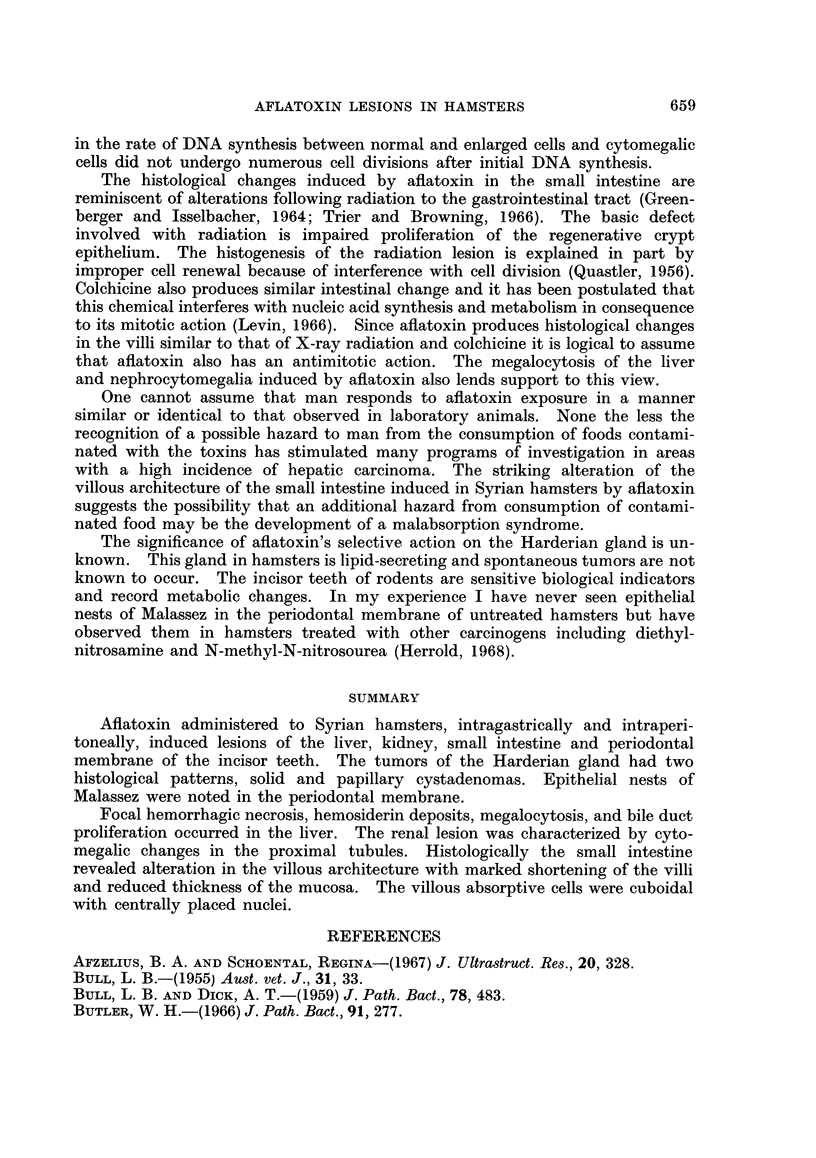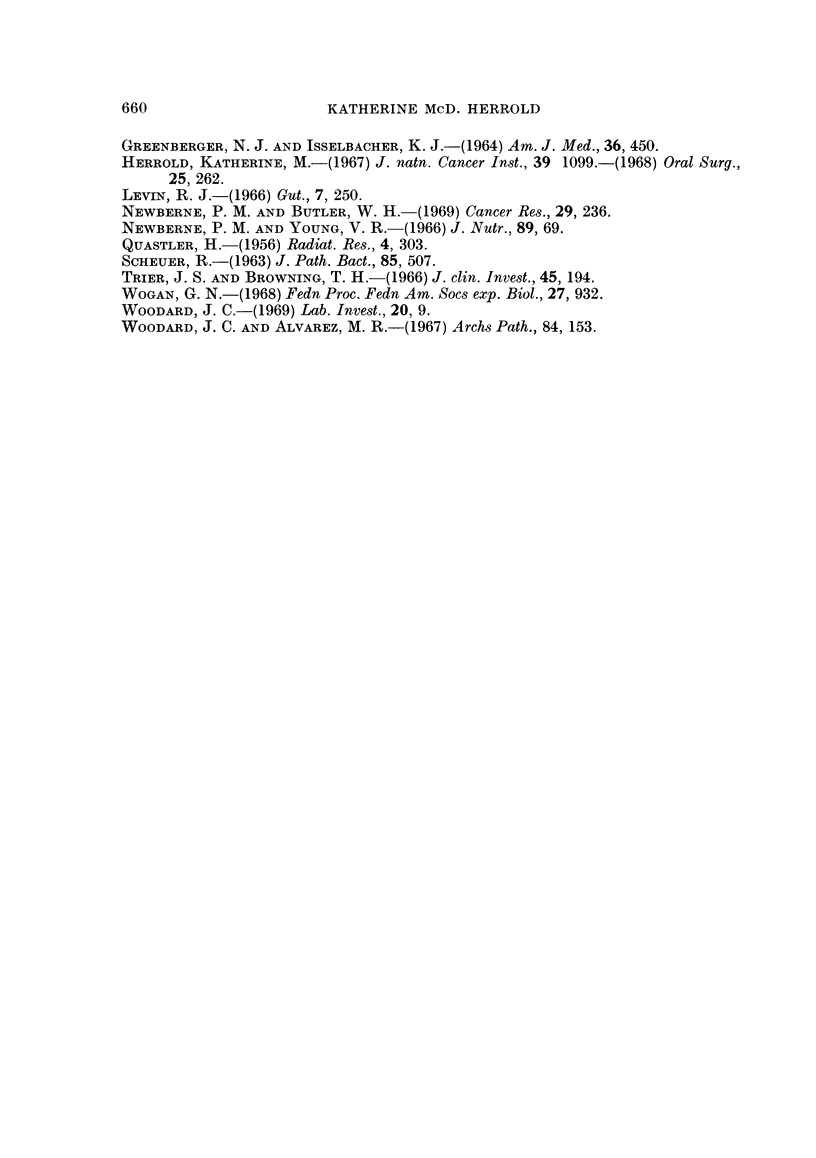# Aflatoxin induced lesions in Syrian hamsters.

**DOI:** 10.1038/bjc.1969.81

**Published:** 1969-09

**Authors:** K. M. Herrold

## Abstract

**Images:**


### AFLATOXIN INDUCED LESIONS IN SYRIAN HAMSTERS

KATHERINE McD. HERROLD

*From the Laboratory of Pathology, National Cancer Institute, Bethesda, Maryland, U.S.A*

Received for publication June 9, 1969.

NEWBERNE AND BUTLER (1969) published a review on the acute and chronic effects of aflatoxin on the liver of domestic and laboratory animals. Their review demonstrated the extensive range of species that are susceptible to the hepatotoxic or hepatocarcinogenic effects of aflatoxin. Although the acute $LD_{50}$ of aflatoxin $B_1$ for hamsters has been reported as 10·2 mg./kg., these authors stated that no information is available on the pathological changes induced. The liver is the principal site for the toxic action in all species but other organs both in the guinea-pig and the rat may be affected by this compound (Butler, 1966).

This report describes the histopathological changes induced in Syrian hamsters by aflatoxin.

### MATERIALS AND METHODS

### *Experimental animals*

Syrian hamsters 1 month of age were used. The hamsters were separated by sex, housed in plastic cages in groups of 5 and fed Purina laboratory chow daily.

### *Aflatoxin*

The aflatoxin was kindly supplied by Dr. W. H. Butler, University College Hospital Medical School, London, W.C.1. A copy of the certificate of analysis of the sample showed the composition to be as follows: $B_1 + B_2$—44 per cent, and $G_1 + G_2$—44 per cent. It was estimated that the amounts of $B_2$ and $G_2$ made up some 2–4 per cent each of the total aflatoxin content.

A 50 per cent ethanol solution of aflatoxin was used for intragastric administration. The aflatoxin was dissolved in a 50 per cent solution of N-N-dimethyl formamide (Eastman Organic Chemicals, Rochester, New York) for intraperitoneal injection. Solutions of the same concentration as the solvents were used for the control groups.

### *Experimental procedure*

There were 2 experimental and 2 control groups each consisting of 10 animals and equally divided according to sex. The aflatoxin and the solvent solutions were administered according to the following schedule:

*Group* 1.—Aflatoxin, intragastric, 0·5 ml. (0·1 mg.) 2 times a week for 10–11 months.

*Group* 2.—Aflatoxin, intraperitoneal, 0·1 ml. (0·2 mg.) weekly for 6–8½ months.

*Group* 3.—50 per cent ethanol solution, intragastric, 0·5 ml., 2 times a week for 10–11 months.

*Group* 4.—50 per cent solution of dimethylformamide (DMF) intraperitoneal, 0·1 ml. weekly for 6–8½ months.

Complete necropsies were performed on all animals killed or found dead. The small and large intestine were each separately prepared using the Swiss roll technique. The tissues were fixed in 10 per cent buffered formalin solution. Paraffin sections cut at 6 $\mu$ were stained by haematoxylin and eosin. Representative sections of the small intestine and Harderian gland were stained with Mayer's mucicarmine, alcian blue periodic acid-Schiff (PAS) and aldehyde fuchsin. Special stains on sections of liver included Gomori's for iron and Stein and Hall's methods for bile pigment.

### RESULTS

*General.*—The localization and frequency of lesions observed in Groups 1 and 2 that received aflatoxin are tabulated in Table I. The organs and tissues involved include the liver, kidney, small intestine, Harderian gland and the periodontal membrane of the incisor teeth. The pathological changes were essentially the same in both groups but the degree and progression of the lesions varied. Similar lesions were not observed in Groups 3 and 4 that received only solvent.

TABLE I.—*Localization and Frequency of Lesions and Tumors Induced by Aflatoxin*

| Group | Number of hamsters | Lifespan (months) | Route of administration | Total dose aflatoxin (mg.) | Lesions: Liver | Kidney | Small intestine | Periodontal membrane | Harderian gland | Adenomas Harderian gland |
|---|---|---|---|---|---|---|---|---|---|---|
| 1 | 10 | 12–30 | Intragastric | 8–10 | 8/10 | 8/10 | 4/10 | 2/10 | 6/10 | 1/10 |
| 2 | 10 | 7–22 | Intraperitoneal | 4·8–7 | 9/10 | 9/10 | 6/10 | 1/10 | 4/10 | 3/10 |

The average life span for Group 1 hamsters was 20 months; for Group 2, 11 months; Group 3, 21 months and Group 4, 19 months. The animals in Groups 1 and 2 failed to gain weight. Some of Group 1 animals after 9 months of treatment and Group 2 animals after 6 months lost weight and this change was more common in females.

### *Histology*

*Liver.*—Two hamsters that received aflatoxin intraperitoneally and died at 7 months of age showed focal areas of hemorrhagic necrosis in the liver. Hemosiderin accumulation was common in animals that died later. The pigment was present both in hepatic and phagocytic cells and most prominent in the periphery of the lobules. The degree of megalocytosis, characterized by the presence of very large hepatocytes, was variable. This change was not observed in every lobule. The islands of enlarged parenchymal cells were usually at the periphery but in some instances the distribution was centrilobular. The megalocytes contain a large nucleus, often pyknotic with coarse chromatin and prominent nucleoli. The cytoplasm is abundant and often finely vacuolated (Fig. 1–2).

The hepatic cords in some lobules were narrowed and the sinusoids dilated. Although the sinusoidal cells were prominent, proliferation was not evident (Fig. 3). There was minimal proliferation of the bile ducts in the portal areas with mild fibrosis and chronic inflammatory cell infiltrates. Cholangitis was occasionally noted. No evidence of regenerative nodules, cirrhosis or tumours of the liver were observed.

*Kidney.*—Although the kidneys appeared normal on gross observation, microscopically there was a striking lesion involving the outer and inner cortex.

This alteration was more prominent and extensive in Group 2 animals that received the aflatoxin intraperitoneally. The renal lesion was characterized by cytomegalic changes in the cells of the proximal tubules. Both the nuclear and cytoplasmic portions of the cells were increased (Fig. 4). In the affected cells, the size, shape of nuclei and condensation of chromatin varied. The nucleoli were prominent, often multiple and the gigantic nuclei had irregular borders (Fig. 5 and 6).

*Small intestine.*—Three animals in each experimental group were killed between 10–16 months of age because of marked weight loss. Grossly the small intestine was either dilated and filled with watery contents or practically empty. The large intestine was contracted and contained no fecal material. No significant gross changes were observed in the intestinal tract of animals that died later but the same histological changes were evident and limited to the small intestine, predominately the jejunum and to a lesser extent the ileum.

Not every villous demonstrated change as some areas revealed relatively normal villi. The epithelial surface remained intact; discontinuities were not observed. The normal delicate villi of the small intestine are lined by a single layer of columnar epithelium with basally located nuclei (Fig. 7). The alteration of the villi in the aflatoxin treated animals was characterized by a decrease in height and increase in width of the villi. No mitotic figures were seen in the crypts of these villi and the submucosal lymphatics were frequently dilated. Not only were the villi shortened but the total thickness of the mucosa was reduced with eventually true villous atrophy (Fig. 8). The cellular infiltrate in the lamina propria, consisting of lymphocytes and plasma cells, was increased in Group 1 animals but essentially within normal limits in Group 2 animals.

The surface epithelium covering the villi showed distinct changes. There was a gradual transition from the tall columnar cells with basal nuclei to cuboidal cells with centrally located nuclei. The cytoplasm of the cells was pale, finely granular and negative for mucin (Fig. 9).

*Harderian gland.*—The lesions consisted of hyperplasia and epithelial atypism of individual acini or portions of a lobule. There was variation in the size and shape of the nuclei with hyperchromatism and prominent nucleoli. Nuclei were situated at various levels in the cells rather than in the usual basal position. The cytoplasm was acidophilic and finely granular.

Tumours of the Harderian gland, depending on their size, presented as a swelling of intraorbital tissue. Microscopically there were two distinct histological patterns which occasionally co-existed in the same gland. Papillary cystadenomas were the most frequent type (Fig. 10) and the homogeneous acidophilic material within the lumens of the cystic spaces gave a negative reaction for mucin. Adenomas of the solid type consisted of compact masses of small cells with oval nuclei and scanty cytoplasm (Fig. 11).

*Periodontal membrane.*—Nests of cells resembling the epithelial nests of Malassez were observed in the periodontal membrane of the lower incisor teeth. The peripheral cells in these oval nests exhibited a tendency to palisade (Fig. 12).

### DISCUSSION

The present study shows that the Syrian hamster is more resistant to the hepatocarcinogenic effect of aflatoxin than the rat. The $B_1$ fraction, believed to be the most carcinogenic, might be effective in inducing liver tumors. The striking enlargement of hepatic cells with pyrrolizidine alkaloids was named by Bull (1955) as megalocytosis. The same reaction occurs in the liver of Syrian hamsters with dimethylnitrosamine (Herrold, 1967). Enlargement of parenchymal cells thus appears to be a feature common to various carcinogens including aflatoxin. Different views have been expressed on the significance of megalocytosis. Bull and Dick (1959) interpret the change as degeneration and Scheuer (1963) suggests that the megalocyte may represent preneoplastic proliferation. The ultrastructural features of enlarged hepatocytes, induced in rats with retrosine, a pyrrolizidine alkaloid, reported by Afzelius and Schoental (1967) are interpreted by these authors as signs of increased metabolic activity. Their autoradiographic studies show that the large parenchymal cells are able to synthesize nucleic acid no less effectively than the smaller ones. These large cells are capable of growth but incapable of division.

The cytomegalic change induced in the kidney by aflatoxin resembles the renal lesion described in rats by feeding diets either marginal in methionine and choline (Newberne and Young, 1966) or diets containing alpha protein, the globulin fraction isolated from soy protein (Woodard and Alvarez, 1967). With these diets only the inner cortex of the kidney is involved and the convoluted portion of the proximal tubule seems to be spared. The aflatoxin induced lesion is present in both portions of the proximal tubule.

The addition of methionine, choline and vitamin $B_{12}$ prevents the development of the pathological kidney change induced by the marginal diet and suggested to Newberne and Young (1966) a possible relationship to methyl group or 1 carbon metabolism. The alpha protein lesion though is not affected by increasing the dietary levels of choline, methionine or vitamin $B_{12}$. Woodard and Alvarez (1967) postulate that alpha protein may contain some toxic substance which exerts a selective action on the kidney. The finding that a variety of food samples including soybeans contain biologically significant levels of aflatoxin (Wogan, 1968) suggests the hypothesis that aflatoxin may be the toxic factor in alpha protein. This seems unlikely though because the renal change with aflatoxin involves both the inner and outer cortex whereas with alpha protein the nephrocytomegalia is limited to the inner cortex. Woodard (1969) detected no difference

---

EXPLANATION OF PLATES

Fig. 1.—Liver showing deposits of hemosiderin and large parenchymal cells. H. and E. × 430.
Fig. 2.—Liver, megalocytes with prominent nucleoli. H. and E. × 380.
Fig. 3.—Narrow hepatic cords with dilated sinusoids and prominence of lining cells. H. and E. × 380.
Fig. 4.—Cytomegalic change in proximal renal tubules. H. and E. × 880.
Fig. 5.—Section of kidney showing cells of normal and intermediate size and megalocyte with prominent nucleoli. H. and E. × 880.
Fig. 6.—Renal tubule lined by cells with gigantic nuclei. H. and E. × 880.
Fig. 7.—Section of small intestine showing normal villous architecture. The nuclei of the tall columnar cells are basally located. H. and E. × 125.
Fig. 8.—Small intestine. Upper, atrophic villi with large cuboidal cells lining surface. Lower, abnormally broad and shortened villi. H. and E. × 125.
Fig. 9.—Flat intestinal mucosa. Note the large surface cells with centrally located nuclei. H. and E. × 340.
Fig. 10.–Papillary cystadenoma of Harderian gland. H. and E. × 108.
Fig. 11.—Harderian gland adenomas, solid and papillary types. H. and E. × 37.
Fig. 12.—Odontogenic epithelial rests of Malassez in periodontal membrane of incisor teeth. H. and E. × 108.

Herrold.

5

6

7

8

Herrold.

9

10

11

12

in the rate of DNA synthesis between normal and enlarged cells and cytomegalic cells did not undergo numerous cell divisions after initial DNA synthesis.

The histological changes induced by aflatoxin in the small intestine are reminiscent of alterations following radiation to the gastrointestinal tract (Greenberger and Isselbacher, 1964; Trier and Browning, 1966). The basic defect involved with radiation is impaired proliferation of the regenerative crypt epithelium. The histogenesis of the radiation lesion is explained in part by improper cell renewal because of interference with cell division (Quastler, 1956). Colchicine also produces similar intestinal change and it has been postulated that this chemical interferes with nucleic acid synthesis and metabolism in consequence to its mitotic action (Levin, 1966). Since aflatoxin produces histological changes in the villi similar to that of X-ray radiation and colchicine it is logical to assume that aflatoxin also has an antimitotic action. The megalocytosis of the liver and nephrocytomegalia induced by aflatoxin also lends support to this view.

One cannot assume that man responds to aflatoxin exposure in a manner similar or identical to that observed in laboratory animals. None the less the recognition of a possible hazard to man from the consumption of foods contaminated with the toxins has stimulated many programs of investigation in areas with a high incidence of hepatic carcinoma. The striking alteration of the villous architecture of the small intestine induced in Syrian hamsters by aflatoxin suggests the possibility that an additional hazard from consumption of contaminated food may be the development of a malabsorption syndrome.

The significance of aflatoxin's selective action on the Harderian gland is unknown. This gland in hamsters is lipid-secreting and spontaneous tumors are not known to occur. The incisor teeth of rodents are sensitive biological indicators and record metabolic changes. In my experience I have never seen epithelial nests of Malassez in the periodontal membrane of untreated hamsters but have observed them in hamsters treated with other carcinogens including diethylnitrosamine and N-methyl-N-nitrosourea (Herrold, 1968).

### SUMMARY

Aflatoxin administered to Syrian hamsters, intragastrically and intraperitoneally, induced lesions of the liver, kidney, small intestine and periodontal membrane of the incisor teeth. The tumors of the Harderian gland had two histological patterns, solid and papillary cystadenomas. Epithelial nests of Malassez were noted in the periodontal membrane.

Focal hemorrhagic necrosis, hemosiderin deposits, megalocytosis, and bile duct proliferation occurred in the liver. The renal lesion was characterized by cytomegalic changes in the proximal tubules. Histologically the small intestine revealed alteration in the villous architecture with marked shortening of the villi and reduced thickness of the mucosa. The villous absorptive cells were cuboidal with centrally placed nuclei.

### REFERENCES

Afzelius, B. A. and Schoental, Regina—(1967) *J. Ultrastruct. Res.*, **20**, 328.
Bull, L. B.—(1955) *Aust. vet. J.*, **31**, 33.
Bull, L. B. and Dick, A. T.—(1959) *J. Path. Bact.*, **78**, 483.
Butler, W. H.—(1966) *J. Path. Bact.*, **91**, 277.

GREENBERGER, N. J. AND ISSELBACHER, K. J.—(1964) *Am. J. Med.*, **36**, 450.
HERROLD, KATHERINE, M.—(1967) *J. natn. Cancer Inst.*, **39** 1099.—(1968) *Oral Surg.*, **25**, 262.
LEVIN, R. J.—(1966) *Gut.*, **7**, 250.
NEWBERNE, P. M. AND BUTLER, W. H.—(1969) *Cancer Res.*, **29**, 236.
NEWBERNE, P. M. AND YOUNG, V. R.—(1966) *J. Nutr.*, **89**, 69.
QUASTLER, H.—(1956) *Radiat. Res.*, **4**, 303.
SCHEUER, R.—(1963) *J. Path. Bact.*, **85**, 507.
TRIER, J. S. AND BROWNING, T. H.—(1966) *J. clin. Invest.*, **45**, 194.
WOGAN, G. N.—(1968) *Fedn Proc. Fedn Am. Socs exp. Biol.*, **27**, 932.
WOODARD, J. C.—(1969) *Lab. Invest.*, **20**, 9.
WOODARD, J. C. AND ALVAREZ, M. R.—(1967) *Archs Path.*, 84, 153.